# Structure and Mechanism of *Staphylococcus aureus* TarS, the Wall Teichoic Acid β-glycosyltransferase Involved in Methicillin Resistance

**DOI:** 10.1371/journal.ppat.1006067

**Published:** 2016-12-14

**Authors:** Solmaz Sobhanifar, Liam J. Worrall, Dustin T. King, Gregory A. Wasney, Lars Baumann, Robert T. Gale, Michael Nosella, Eric D. Brown, Stephen G. Withers, Natalie C. J. Strynadka

**Affiliations:** 1 Department of Biochemistry and Center for Blood Research, University of British Columbia, Vancouver, British Columbia, Canada; 2 Department of Chemistry, University of British Columbia, Vancouver, British Columbia, Canada; 3 Department of Chemistry and Biomedical Sciences, McMaster University, Hamilton, Ontario, Canada; National Jewish Health, UNITED STATES

## Abstract

In recent years, there has been a growing interest in teichoic acids as targets for antibiotic drug design against major clinical pathogens such as *Staphylococcus aureus*, reflecting the disquieting increase in antibiotic resistance and the historical success of bacterial cell wall components as drug targets. It is now becoming clear that β-O-GlcNAcylation of *S*. *aureus* wall teichoic acids plays a major role in both pathogenicity and antibiotic resistance. Here we present the first structure of *S*. *aureus* TarS, the enzyme responsible for polyribitol phosphate β-O-GlcNAcylation. Using a divide and conquer strategy, we obtained crystal structures of various TarS constructs, mapping high resolution overlapping N-terminal and C-terminal structures onto a lower resolution full-length structure that resulted in a high resolution view of the entire enzyme. Using the N-terminal structure that encapsulates the catalytic domain, we furthermore captured several snapshots of TarS, including the native structure, the UDP-GlcNAc donor complex, and the UDP product complex. These structures along with structure-guided mutants allowed us to elucidate various catalytic features and identify key active site residues and catalytic loop rearrangements that provide a valuable platform for anti-MRSA drug design. We furthermore observed for the first time the presence of a trimerization domain composed of stacked carbohydrate binding modules, commonly observed in starch active enzymes, but adapted here for a poly sugar-phosphate glycosyltransferase.

## Author Summary

Historically, β-lactam class antibiotics such as methicillin have been very successful in the treatment of bacterial infections, effectively destroying bacteria by rupturing their cell walls while posing little harm to the human organism. In recent years, however, the alarming emergence of Methicillin Resistant *S*. *aureus* or MRSA has resulted in a world-wide health crisis, calling on new strategies to combat pathogenesis and antibiotic resistance. As such, understanding the pathways and players that orchestrate resistance is important for overcoming these mechanisms and restoring our powerful β-lactam antibiotic arsenal. In this article we describe the crystal structure of TarS, an enzyme responsible for the glycosylation of wall teichoic acid polymers of the *S*. *aureus* cell wall, a process that has been shown to be specifically responsible for methicillin resistance in MRSA. TarS is therefore a promising drug target whose inhibition in combinational therapies would result in MRSA re-sensitization to β-lactam antibiotics. Here we present the first structure of TarS together with several snap-shots of its substrate/product complexes, and elucidate important catalytic features that are valuable for rational drug design efforts to combat resistance in MRSA.

## Introduction

Methicillin-Resistant *Staphylococcus aureus* (MRSA) is a leading cause of life-threatening nosocomial infections including pneumonia, bacteremia, and surgical wound infections [1]. Due to wide-spread β-lactam antibiotic resistance, the first-line treatment for serious MRSA infections has been vancomycin, a glycopeptide class antibiotic. However rising resistance to vancomycin has forced the use of undesirable alternatives with high cost and dose limitations due to adverse events [2]. Both β-lactam and glycopeptide antibiotics disrupt peptidoglycan cross-linking that eventually weakens the integrity of the bacterial cell wall and leads to lysis. Due to the efficacy and safety profile of β-lactam antibiotics, re-sensitization of MRSA to these drugs is a promising option that entails understanding of complex resistance mechanisms. Resistance in MRSA mainly evolves from the expression of PBP2a, a β-lactam-insensitive penicillin-binding protein that can cross-link peptidoglycan in the presence of clinically relevant concentrations of nearly all β–lactam antibiotics (reviewed in [3]). Interestingly, recent reports have uncovered the role of wall teichoic acids and more specifically, their β-O-GlcNAc decorations, in mediating MRSA resistance to β-lactams [4,5], opening new avenues for drug discovery efforts aimed at re-sensitization.

Teichoic acids are anionic glycopolymers that compose an astonishing 60% of the dry weight of the cell wall in Gram-positive bacteria [6]. These polymers may either be attached to membranes in the form of lipoteichoic acids (LTAs) or transferred onto peptidoglycan as wall teichoic acids (WTAs). Collectively, TAs are implicated in diverse processes such as coping with environmental stress [7,8], interaction with receptors and biomaterials [9,10], induction of inflammation [11–13], phage binding [14,15], immune evasion [16], biofilm formation [17], resistance to lysozyme [18], and resistance to antimicrobial molecules [5,19–21]. This adaptability arises largely from D-alanylation and glycosylation of TA polyol hydroxyl groups, influencing the physical and interactive properties of the cell wall. In most *S*. *aureus* strains, WTAs consist of polyribitol phosphate (polyRboP) chains of 40–60 repeats that are attached to the peptidoglycan via a disaccharide linkage unit to C6 hydroxyls of occasional N-acetylmuramic acid residues [22]. The C4 hydroxyls of *S*. *aureus* WTAs are furthermore heavily substituted with N-acetylglucosamine (GlcNAc) via α- or β-O-linkages. The configuration of the glycosidic linkage varies according to strain, with some having exclusive α- or β-O-linked GlcNAc, and others displaying a mixture [23,24]. In *S*. *aureus*, WTA GlcNAcs serve as receptors for phage binding [15], have long been recognized as important antigens in the host-antibody response [25–27], and have more recently been implicated in biofilm formation [28]. Furthermore, the stereochemistry of GlcNAc glycosidic linkages appears to directly influence both the biology and pathogenicity of *S*. *aureus* and other Gram-positive bacteria on a strain-specific level. The enzymes responsible for *S*. *aureus* WTA GlcNAcylation are the α-glycosyltransferase TarM and the β-glycosyltransferase TarS. Both these enzymes reside in the cytoplasm and decorate nascent WTA chains before transport and attachment to the peptidoglycan sacculus. Of significance is the recent discovery that β-O-GlcNAcylation of *S*. *aureus* WTA is specifically responsible for methicillin resistance in MRSA, which may be due to the possible direct or indirect recruitment of the β-lactam insensitive PBP2a that mediates resistance[5,29]. Accordingly, the deletion of TarS has been shown to result in the re-sensitization of MRSA strains to β-lactam antibiotics [5]. TarS mediated WTA β-O-GlcNAcylation has also been implicated in the induction of anti-WTA IgG-mediated complement activation and opsonophagocytosis in clinically isolated *S*. *aureus* strains [30]. We have recently published the first structure of TarM and elucidated its catalytic mechanism [31]. In this report, we present the first structure of TarS in the presence of donor substrate UDP-GlcNAc, elucidate various features involved in catalysis, and describe a novel trimerization domain composed of tandem carbohydrate binding motifs. Due to the pivotal role of TarS in MRSA resistance, its structure is particularly valuable for rational drug design efforts in combination therapies aimed at MRSA re-sensitization.

## Results

### Overall structure of TarS

**Fig 1:**
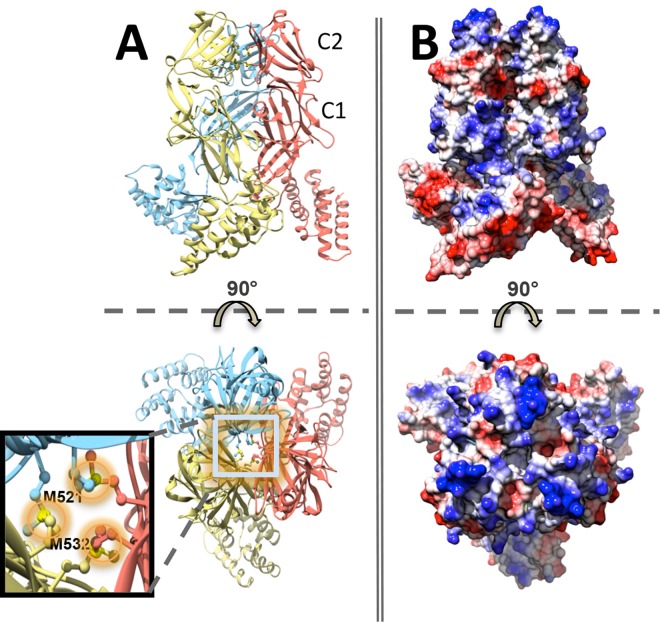
Structural features of TarS_217-573_. (A) Ribbon representation of TarS_217-573_ in two views related by a 90° rotation along the x-axis, where each monomer is indicated by color. The bottom view displays methionine residues that cluster among monomers. (B) Electrostatic surface representation of TarS_217-573_ in two views related by a 90° rotation along the x-axis, in the same orientation as (A).

**Fig 2:**
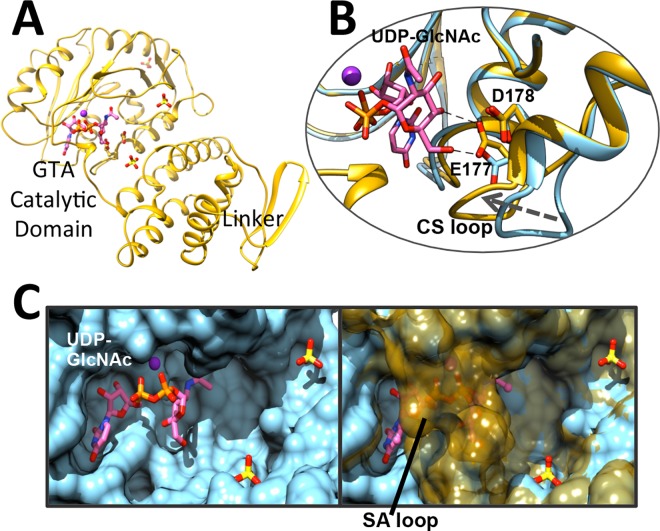
Structural features of TarS_1-349._ (A) Ribbon representation of TarS_1-349_ in complex with UDP-GlcNAc (pink), Mn^2+^ (purple sphere) and sulfates (yellow). (B) Comparison of the position of the CS loop in the superimposed UDP complexed (blue) and UDP-GlcNAc complexed (gold) ribbon structures. (C) Comparison of the disordered and ordered states of the SA loop in surface representations of the UDP complexed (blue) structure (left) and superimposed UDP complexed and UDP-GlcNAc complexed (50% transparent, gold) structures (right) highlighting the partial occlusion of the active site in the ordered state. Only UDP-GlcNAc is displayed for simplicity. Substrates and residues are displayed in stick form and colored according to heteroatom type.

**Fig 3:**
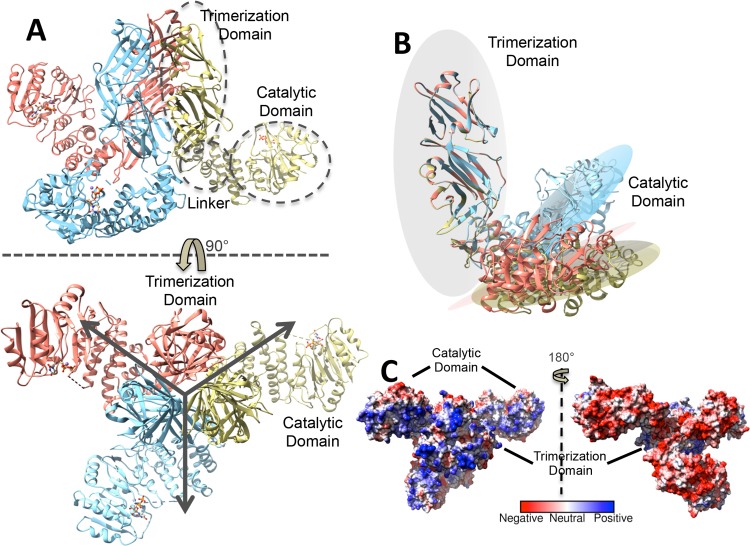
Structural features of full-length TarS. (A) Ribbon representation of TarS in complex with UDP (displayed in ball and stick form and colored according to heteroatom type) in two views related by a 90° rotation along the x-axis. Each monomer is indicated by color, and the catalytic and trimerization domains are indicated in the top view. Arrows in the bottom view demonstrate the 3-fold symmetry in the trimerization domain. (B) Overlay of TarS monomer trimerization domains displaying the relative positions of corresponding catalytic domains. The superimposed trimerization domain is represented by a light gray plane and the catalytic domains by colored planes corresponding to (A). (C) Electrostatic surface representation of TarS in two views related by a 180° rotation along the y-axis, with the left view in the same orientation as the bottom view in (A).

The structures presented here are of the TarS full-length protein (1–573), the TarS_1-349_ (1–349) construct consisting of the catalytic domain (1–319) and the linker (320–352), and the TarS_217-573_ (217–573) construct consisting of the catalytic domain C-terminal helical bundle (217–319), the linker and the trimerization domain (353–573) (S1 Fig). Refinement statistics of the final structural models are presented in Table 1. The full-length TarS crystals diffracted with strong anisotropy and a high resolution limit of 4 Å was applied. Due to the limited structural resolution of full-length TarS, efforts were made to obtain higher resolution structures of the individual protein domains. Limited (thermolysin) proteolysis of the purified full-length TarS protein resulted in crystals diffracting to ~ 2.3 Å resolution, with SAD phasing using an iodide derivative revealing cleavage of the N-terminal region of the catalytic domain (1–216) and the structure of the TarS_217-573_ as described (Fig 1). TarS_1-349_, described above, was isolated by designing a structure-guided truncation mutant (based on the TarS_217-573_ structure) that resulted in a structure at 2.3 Å resolution with intact UDP-GlcNAc bound in the active site (Fig 2A). The intact (rather than hydrolyzed) UDP-GlcNAc in the active site was achieved by soaking crystals in increased concentrations of substrate (50 mM) before freezing. Native and UDP bound structures were also obtained to similar resolution (S2 Fig). The higher resolution TarS_1-349_ and TarS_217-573_ structures were subsequently used as molecular replacement search models to solve the structure of the full-length TarS, revealing a “hanging basket” like structure (Fig 3A) with variation in the relative domain orientation of the three catalytic domains with respect to the trimerization domain (Fig 3B). TarS furthermore displays a pronounced electrostatic sidedness, which may be related to membrane localization or substrate/partner interactions (Fig 3C). In light of the data quality, refinement was closely monitored using the higher resolution structures as restraints and validated by the visualization of OMIT maps for both protein (S3 Fig) and ligands (S2C Fig). Although TarS was co-crystallized in the presence of the UDP-GlcNAc sugar donor, only the cleaved UDP product seemed to be observed (ligand mFo-dFc simulated annealing omit electron density shown in S2C Fig) and the crystals were too sensitive to withstand soaking in higher concentrations of UDP-GlcNAc as for the TarS_1-349_ crystals. The overlap of the TarS_1-349_ and TarS_217-573_ structures with regard to the C-terminus of the catalytic domain and the linker region (217–349) further allowed us to superimpose these domains relative to each other and to the lower resolution full-length structure, providing us with essentially high-resolution views for the entire span of the TarS structure.

**Table 1:** Data Collection, phasing and refinement statistics

Construct	Full-length UDP	TarS_217-573_	TarS_1-349_ Native	TarS_1-349_ UDP	TarS_1-349_ UDP-GlcNAc	TarS_1-349_ UDP-GlcNAc No Mn^2+^
**Data collection**						
Space group	P21	P32	C2	P21	P1	P1
Cell dimensions						
*a*, *b*, *c* (Å)	71.85 191.47 97.03	105.66 105.66 80.48	98.66 58.66 86.68	53.07 60.19 65.39	57.51 57.58 86.48	57.002 57.37 86.786
α,β,γ (°)	90 109.5 90	90 90 120	90 97.01 90	90 101.51 90	83.81 82.92 61.88	83.027 84.553 62.262
Wavelength (Å)	1.000	0.979	1.000	1.542	1.000	0.984
Resolution (Å)	35.90–4.0 (4.38–4.0)	52.83–2.31 (2.39–2.31)	42.29–2.3 (2.38–2.3)	28.28–2.22 (2.30–2.22)	31.43–2.33 (2.41–2.33)	48.56–1.9 (1.97–1.9)
*R*_sym_	0.123 (0.665)	0.1409 (1.013)	0.05811 (0.3529)	0.04976 (0.2)	0.07954 (0.4743)	0.06231 (0.7734)
*CC1/2*	0.99 (0.796)	0.998 (0.644)	0.998 (0.932)	0.999 (0.959)	0.993 (0.622)	0.997 (0.448)
*I* / σ*I*	8.0 (2.1)	18.14 (2.02)	15.88 (4.00)	23.09 (8.61)	9.91 (2.10)	8.78 (1.12)
Completeness	0.99 (0.99)	0.98 (0.94)	0.97 (0.98)	1.00 (0.96)	0.95 (0.96)	0.95 (0.94)
Redundancy	3.5 (3.5)	10.7 (6.9)	3.7 (3.9)	4.9 (4.5)	2.0 (2.0)	2.0 (2.0)
**Refinement**						
Resolution (Å)	4.0	2.31	2.3	2.22	2.33	1.9
No. reflections	20792 (4940)	22767 (2134)	21511 (2131)	19997 (1944)	39577 (3975)	71900 (7140)
*R*_work_ / *R*_free_	0.280/0.306	0.223/0.268	0.214/0.253	0.176/0.218	0.189/0.233	0.189/0.226
No. atoms						
Protein	13620	2916	2738	2744	5668	5674
Ligand/ion	0	5		46	100	78
Water	0	181	163	173	319	364
*B*-factors						
Protein	185.42	54.10	51.23	31.88	42.64	38.50
Ligand/ion	n/a	52.75	n/a	44.76	41.47	40.56
Water	n/a	43.84	46.10	39.46	40.17	41.71
R.m.s deviations						
Bond lengths (Å)	0.003	0.004	0.005	0.008	0.004	0.014
Bond angles (°)	0.62	0.7	0.69	1.01	0.66	1.18

**Fig 4:**
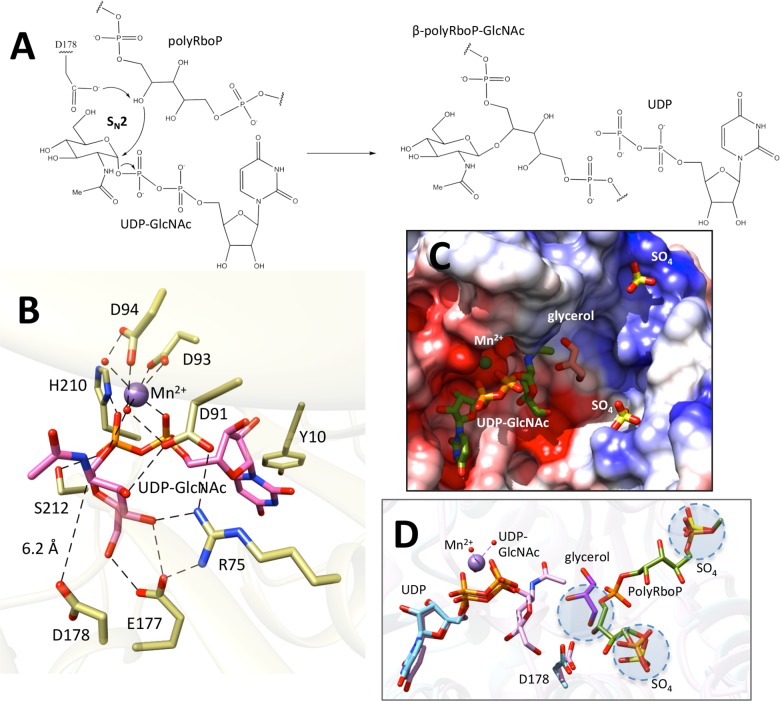
Catalytic features of TarS. (A) Schematic of the proposed S_N_2 reaction mechanism, with the glycosyltransferase reaction between UDP-GlcNAc and polyRboP resulting in UDP and β-polyRboP-GlcNAc. (B) Close-up of the catalytic site showing interactions of active site residues (green) with UDP-GlcNAc (pink), Mn^2+^ (purple) and ordered waters (red). Interactions between atoms are displayed by dotted lines, and the distance between the C1 position of GlcNAc and the hydroxyl of the proposed catalytic base (D178) is indicated. (C) Electrostatic surface representation of the TarS active site in complex with UDP-GlcNAc (green), sulfates (yellow), and glycerol (pink) (superimposed from SpsA (PDB:1qgq)). (D) Docking of a PRboPRboP molecule (green) in the TarS active site. The model places an RboP C4 hydroxyl in close proximity to the catalytic D178, as well as a glycerol (purple) hydroxyl superimposed from the SpsA structure (circled). The terminal phosphates of PRboPRboP furthermore superimpose closely with sulfates bound in the structure (circled). The positions of UDP (blue) and UDP-GlcNAc (pink) are indicated. Ligands are displayed in stick form and colored according to heteroatom type.

TarS possesses a catalytic domain with a canonical GTA fold (one of two distinct A/B folds characteristic of nucleotide-sugar dependent glycosyltransferases) consisting of two closely associated α/β/α sandwich Rossmann motifs that abut and form a continuous central β-sheet (Fig 2A and S1 Fig). According to the Carbohydrate-Active Enzymes (CAZY) database that classifies enzyme families based on sequence homology [32], TarS belongs to the GT2 family that includes a large (and evolutionarily ancient) group of GTA fold stereochemistry-inverting enzymes acting on a variety of substrates, many of which are polysaccharides (e.g. cellulose, chitin, hyaluronic acid, etc.). TarS possesses a D(91)XDD motif with aspartates coordinating a metal cation, a feature that is typical of the GTA superfamily (Fig 4B) [33]. The structures of native and UDP bound TarS_1-349_ were similar to that obtained for the lower resolution UDP bound full-length TarS (overall main chain root mean squared deviation (rmsd) of 0.56 Å over 335 atom pairs), with minor differences in the linker region (320–349). Brief soaking of the TarS_1-349_ crystals with UDP-GlcNAc resulted in an enzyme-donor complex with UDP-GlcNAc trapped in the active site as evidenced by the ligand mFo-dFc simulated annealing omit electron density (S2C Fig). This structure displayed differences in two key loop regions that appear to be important for catalysis (discussed below; Fig 2B and 2C).

Two C-terminally localized regions in the TarS_217-573_ structure (encapsulating the trimerization domain), designated here as C1 (353–495) and C2 (496–573) (S1 Fig), were also observed, where a series of β-sheets of unique sequence participate in an extensive trimerization interface (buried surface area: 6970 Å^2^, predicted as stable by PISA [34]) and connect to a linker composed of 2 anti-parallel β-strands and an α-helix that leads into the catalytic domain (Fig 1A and S4 Fig). These C-terminal tandem domains assume an immunoglobulin-like fold typical for starch binding domains, and show close structural resemblance (but low sequence identity ~ 18%) to the N-terminal domains (N1 and N2) of *Anoxybacillus sp*. LM18-11 pullulanase [35], with N1 (10–88) corresponding to TarS C2 (overall main chain rmsd of 1.0 Å over 25 atom pairs) and N2 (110–186) corresponding to TarS C1 (overall main chain rmsd of 1.2 Å over 36 atom pairs). Interestingly, the tandem domains of the two enzymes are inverted with respect to their corresponding catalytic domains, existing C-terminally in TarS and N-terminally in pullulanase. Nevertheless the order of the domains is maintained, with C1/N2 adjacent to the catalytic domain, followed by C2/N1 (S4 Fig). The N2 domain is highly conserved among pullulanases and belongs to carbohydrate binding module (CBM) family CBM48 that typically binds pullulan and glycogen. The N1 domain is located at the highly variable N-terminus characteristic of pullulanases and is observed in complex with maltotriose and maltotetraose, classifying it to a novel CBM68 family [35], supporting the possibility that the analogous TarS trimerization domain could participate in binding the teichoic acid glycopolymer. Furthermore, prominent basic grooves along the surface of the trimerization domain, and notably along the C1 domain leading into the active site (Fig 1B and Fig 4C), suggest that both C1 and C2 domains likely participate in polyRboP binding. Another interesting feature is the close proximity (4.0 Å) of methionines (M521 and M532) from each of the 3 different monomers at the bottom surface of the trimerization interface, forming a hexameric methionine cluster that we hypothesize may be involved in promoting plasticity/adaptability of TarS during multivalent interactions with substrates or potential cell-wall partners (Fig 1A).

Altogether, TarS features a novel architecture with closely-associated trimerization domains connected by a linker region to three protruding catalytic domains that face away from each other (Fig 3A). The physiological relevance of the trimeric architecture is supported by size exclusion chromatography-multiangle light scattering (SECMALS) with an elution profile that corresponds predominantly to a 200 kDa species (theoretical monomer molecular weight is 66 kDa) (S5 Fig). Furthermore, structure-guided mutants aimed at disrupting the trimerization interface (M521R, M532R) (Fig 1A) resulted in the expression of insoluble protein aggregates. The structure of full-length TarS suggests motion between the catalytic domains and corresponding trimerization domains, where an overlay of trimer monomers reveals varying angles (50° vs. 64° vs. 78° as measured with the UCSF Chimera package [36]) between the common plane of the trimerization domain and the individual planes of the catatlytic domains, with the hinge point centered on P352 (Fig 3B and S1 Movie). The difference in angles is unlikely to have resulted from crystal packing artifacts, given that the catalytic domains in the full-length structure are not involved in any crystal contacts. We propose therefore that the apparent flexibility between the two domains may be related to substrate binding and/or processivity.

### Catalytic mechanism

Our analysis of the enzymatic mechanism of TarS was facilitated by the capture of various TarS_1-349_ structures encapsulating the catalytic domain in complex with metal and substrates. These include the native structure with bound Mn^2+^ (free enzyme), the UDP-GlcNAc bound structure in the presence and absence of Mn^2+^ (the binary Michaelis complex), and the UDP bound structure with Mn^2+^ (product complex) (S2 Fig). The identity of the cation as Mn^2+^ is inferred from the observation that if Mn^2+^ is not specifically added during crystallization, the corresponding electron density is absent. Furthermore, inductively coupled plasma mass spectrometry (ICP-MS) studies revealed that TarS, purified in buffers without cation, lacked a bound metal, making it unlikely that another cation would have been carried through to crystallization. This data would further suggest that the cation does not play a structural role, but rather a functional role in the enzyme. The above structures show notable differences in two key loops, depending upon the presence or absence of the intact UDP-GlcNAc donor substrate. The first loop (171–178), designated as the catalytic site (CS) loop, contains the putative base catalyst D178 and moves towards the catalytic center in the presence of the intact UDP-GlcNAc donor substrate (Fig 2B). The second loop (205–215), designated as the substrate access (SA) loop, is ordered only in the presence of UDP-GlcNAc and sterically occludes an otherwise open channel leading into the active site in the absence of the intact donor (Fig 2C). Based on these observations it may be inferred that in the native structure, the SA loop is disordered allowing binding of UDP-GlcNAc, upon which the CS loop moves closer to the active site center and the SA loop becomes ordered, occluding the active site channel. This occlusion may serve to exclude water in order to decrease background hydrolysis, and/or to possibly guide the correct positioning of the acceptor polyRboP by providing a restricted passage for entry and facilitating binding. Both these loops contain several active site residues that are essential for catalysis as described below. Upon UDP-GlcNAc cleavage, where only UDP remains bound to the enzyme, the SA loop is once again disordered, likely allowing the release of the leaving group and subsequent binding of a new UDP-GlcNAc donor substrate. Similar adaptability is witnessed in active site proximal loops of many diverse GTA class glycosyltransferases, including as examples the GTPase glycosyltransferase TcdA [37], the ABO(H) group A and B glycosyltransferases GTA and GTB [38], and the maltosaccharide synthase glycogenin [39], reflecting the plasticity required for binding and catalysis involving a wide range of donor/acceptor substrates. The signature DXD motif of TarS is believed to be critical for divalent cation coordination and catalysis. The two aspartates within this motif are D91 and D93, situated adjacent to a third aspartate, D94, whose role in catalysis was also investigated. Structural analysis revealed that Mn^2+^ is coordinated in a hexahedral manner, via a bivalent interaction with both side chain carboxylate oxygens of D93, the UDP-GlcNAc/UDP diphosphates O1A and O1B, as well as two ordered water molecules. The R206 guanidinium group furthermore forms a hydrogen bond with the D93 carboxylate side chain and likely ensures its proper orientation. D94 has an indirect role in that its side chain carboxylate forms hydrogen bonds with the two Mn^2+^ coordinating waters. The D91 side chain carboxylate also forms a hydrogen bond with one of the waters that coordinates Mn^2+^. This divalent metal and its coordinating waters, along with several active site residues, in turn contribute to binding of the UDP-GlcNAc donor substrate (Fig 4B). Notably the side chain carboxylate oxygen of E177, located in the SA loop, moves into position to form hydrogen bonds with the C4 and C6 GlcNAc hydroxyls (Fig 4B and Fig 2B), the side chain imidazole of H210 and hydroxyl of S212, located in the CS loop, form stabilizing interactions with the O1B and O2B phosphates when ordered upon UDP-GlcNAc binding, and the uracil ring of UDP-GlcNAc is stabilized by offset π-π interactions with the Y10 phenol side chain (Fig 4B). Interestingly, no stabilizing interactions are observed with the N-acetyl group of GlcNAc, such that specificity may extend to similar sugars and may be of interest for inhibitor design. This observation is in agreement with the previous finding that TarS can use UDP-Glc as an alternative donor substrate, suggesting tolerance at the C2 position [5]. A key residue that appears to bridge the CS and SA loops is R75, whose side chain guanidinium forms interactions with D91 and E177 carboxylates as well as the C4 hydroxyl of GlcNAc (Fig 4B). Interestingly, TarS has also been shown to use UDP-GalNAc as a substrate, although far less efficiently than UDP-Glc or UDP-GlcNAc [5]. This suggests a lesser degree of tolerance at the C4 position, reflecting the interactions of E177 and R75 with the C4 GlcNAc hydroxyl.

GTA inverting glycosyltransferases are believed to adopt a concerted S_N_2-like displacement mechanism for catalysis, as supported by structural studies [40–43], hybrid quantum mechanical/molecular mechanical studies [44,45], and kinetic isotope effect measurements [46,47]. The active site conformation of TarS appears to also adhere to an S_N_2-type reaction mechanism, according to which the teichoic acid acceptor molecule is activated by general base-catalysed abstraction of the polyRboP C4 hydroxyl group hydrogen in concert with nucleophilic attack at the β-face of the UDP-GlcNAc C1 anomeric centre (Fig 4A). Departure of the leaving UDP is stabilized by the coordinating Mn^2+^ and leads to glycosyl transfer with inversion of the stereochemistry of the GlcNAc anomeric centre. UDP-GlcNAc itself is furthermore observed to assume a “tucked under” conformation (as common in other glycosyltransferases [38,48–55]), where the GlcNAc sugar is tucked below the plane of the UDP diphosphates, allowing exposure of the scissile bond to nucleophilic attack (Fig 4B). In TarS, D178, situated 6.2 Å away on the β-face of the C1 anomeric carbon, is suitably positioned to act as a Brønsted base catalyst for the incoming acceptor (Fig 4B), and shows good spatial agreement with the catalytic aspartate of SpsA [56], a prototypical inverting GTA from the GT2 family (sharing 28% identity with TarS), when the structures are superimposed (overall main chain rmsd of 1.1 Å over 107 atom pairs) (S6A Fig). The Mn^2+^ that ligands D93 is also observed to closely superimpose with the equivalent D99 of the Mn^2+^ dependent SpsA. Indeed, a glycerol molecule trapped in the SpsA structure in proximity to the catalytic aspartate could, by analogy, provide a clue as to the positioning of the incoming polyRboP in the TarS structure (S6B Fig). Trapped sulfates from our crystallization condition were also observed in the TarS_1-349_ structure along two basic grooves leading into the active site centre, potentially indicating where the phosphates of the acceptor polyRboP may be bound (Fig 2A, Fig 4C). In support of this, we used AutoDock Vina to model a PRboPRboP molecule in the TarS active site using a highly exhaustive search protocol. The top 20 scoring poses reveal that the terminal phosphates of PRboPRboP overlap closely with the two sulfates situated in the basic grooves discussed above. A RboP C4 hydroxyl in the lowest energy pose was furthermore situated in close proximity to the catalytic D178, and was oriented similarly to a glycerol hydroxyl superimposed from the SpsA structure (Fig 4D). Brown et al. have previously shown TarS to be exclusive and highly specific for its polyRboP acceptor, having tested various alternate acceptor substrates including RboP, polyGroP-WTA, LTA, and CDP-ribitol [5]. Based on our model, it is possible that phosphate binding sites residing in basic grooves along the protein surface could determine the acceptor specificity by dictating the distance between adjacent phosphates, such that the shorter polyGroP unit chains may be incompatible with binding. Binding of multiple polyol phosphate units along the surface could also collectively increase the substrate binding affinity, allowing greater selectivity for the polymeric substrate over similar “monomer” substrates such as RboP and CDP-ribitol.

**Fig 5:**
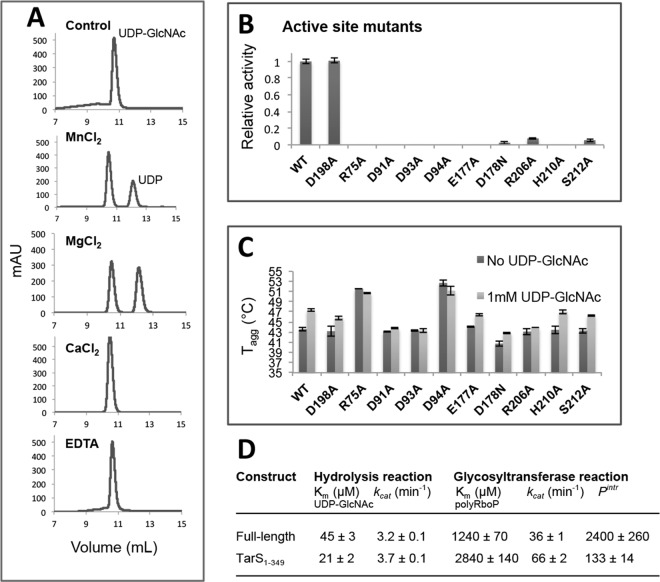
Analysis of TarS catalytic activity. (A) Comparison of activity in the presence and absence of divalent cations by HPLC based UDP detection. A no protein control was included for reference, and reactions proceeded in the presence of 1mM UDP-GlcNAc and 1mM metal/EDTA where indicated. (B) Relative activities of various TarS catalytic site mutants compared to wild-type by HPLC based UDP detection in the presence of 1mM UDP-GlcNAc. For comparative purposes, relative activity is given as a fraction of wild-type activity whose value was adjusted to 1.0. (C) Thermostability of various TarS catalytic site mutants in the presence and absence of UDP-GlcNAc, analyzed by differential static light scattering as a measure of T_agg_ upon thermodenaturation. (D) Kinetic parameters of full-length and TarS_1-349_ constructs. Kinetic parameters were determined using continuous fluorescence-based UDP detection with increasing UDP-GlcNAc concentrations (hydrolysis reaction) or increasing polyRboP concentrations (glycosyltransferase reaction; in presence of 1mM UDP-GlcNAc).

To analyze the glycosyltransferase activity of TarS, polyRboP was isolated from the cell wall of *S*. *aureus* strain RN4220 and attached GlcNAcs were hydrolytically cleaved with α- and β-N-acetylglucosaminidases (NAGLUs) to liberate free acceptor sites. PolyRboP was then purified on a DEAE weak anion exchange column to remove NAGLUs and analyzed by ICP-MS to determine, according to the measured phosphate concentration, the molar concentration of constituent single RboP units in the WTA chain. TarS activity on this substrate was then analyzed by both direct HPLC-based and indirect fluorescence-based UDP detection methods. The HPLC method was used to test for the presence of activity, where a TSKgel DEAE-5PW weak anion exchange column was used to separate UDP-GlcNAc from released UDP upon donor hydrolysis. Using this method, the activity of wild-type TarS and its metal dependency was confirmed (Fig 5A). We note that TarS displayed some promiscuity towards Mn^2+^ and Mg^2+^, showing activity in the presence of both, with Mg^2+^ resulting in a higher level of UDP-GlcNAc hydrolysis. The presence of Ca^2+^ however did not result in observable activity (Fig 5A). Several constructed active-site mutants were also tested for activity, along with a designed control (D198A) chosen distal from the catalytic center (Fig 5B). Based on these results, mutations R75A, D91A, D93A, D94A, E177A, and H210A abolished activity as defined by UDP-GlcNAc hydrolysis, whereas mutations D178N, R206A, and S212A led to severe decreases in activity, validating the importance of these residues for catalysis, as discussed above. These mutants were also screened for thermostability, showing that, except for R75A and D94A, which were actually more stable than wild-type, the mutations did not affect overall protein stability (Fig 5C). Analysis of the thermostability data also revealed that mutants E177A, D178N, H210A, and S212A, residing in flexible CS and SA loops, were stabilized in the presence of UDP-GlcNAc similar to the wild-type enzyme. This suggests that these residues may play only an ancillary role in initial substrate binding, consistent with the unfavorable positioning and disorder of respective CS and SA loops prior to UDP-GlcNAc binding. Mutants R75A, D91A, D93A, D94A, and R206A however showed no significant stabilization in the presence of UDP-GlcNAc, suggesting that these mutations are detrimental for UDP-GlcNAc binding and further emphasizing the roles of these residues in donor substrate interactions, as observed structurally (Fig 4B). For kinetic measurements, continuous fluorescent monitoring of UDP release was achieved with the ADP Quest Assay kit (Discover Rx), and kinetic parameters for both wild-type (full-length) and TarS_1-349_ (lacking the trimerization domain) constructs were determined (Fig 5D). Although TarS has been previously shown unable to GlcNAcylate single RboP units [5], we found that the reaction could be driven under high RboP concentrations. Therefore, the glycosyltransferase activity of TarS was further confirmed by mass-spectroscopic identification of the RboP-GlcNAc reaction product of UDP-GlcNAc and RboP (S7 Fig). Kinetic parameters for wild-type and TarS_1-349_ constructs were similar, indicating that the trimerization domain appears to be dispensable for UDP-GlcNAc hydrolysis activity. Furthermore, for both constructs, the glycosyltransferase activity *k*_*cat*_ in the presence of polyRboP was more than 10 fold greater than the hydrolysis activity in its absence. However, there were some small differences. For instance the K_m_ (UDP-GlcNAc; 21 ± 2 μM) of the TarS_1-349_ hydrolysis reaction was somewhat lower than that of the wild-type (45 ± 3 μM). In addition, both the K_m_ (polyRboP; 2840 ± 140 μM)) and *k*_*cat*_ (66 ± 2 min^-1^) of the TarS_1-349_ glycosyltransferase reaction were somewhat higher than those of the wild-type (K_m_ 1240 ± 70 μM; *k*_*cat*_ 36 ± 1 min^-1^)). These differences may be associated with the trimerization domain’s increasing of polyRboP binding affinity and its possible effect on the relative orientation (suggested structurally) and local concentration of the linked catalytic domain that could influence the reaction. Nevertheless, the second order rate constants *k*_*cat*_/K_m_ for both full-length and TarS_1-349_ glycosyltransferase reactions are very similar, suggesting the trimerization domain does not have a large role in catalysis, but is more likely involved in other events such as protein interactions and/or processivity. In order to examine whether TarS is a processive enzyme (i.e. catalyzes multiple rounds of reactions before substrate dissociation [57]) and the possible involvement of the trimerization domain in processivity, we measured association and dissociation rate constants (*k*_*on*_ and *k*_*off*_) of wild-type and TarS_1-349_ using biolayer interferometry (S8 Fig). Intrinsic processivity (theoretical potential for processivity: *P*^*intr*^) was then calculated using the approximation *P*^*intr*^ ~ *k*_*cat*_ /*k*_*off*_ (where *k*_*cat*_ pertains to glycosyltransferase activity) as previously described [58,59] (Fig 5D). The results show that although TarS_1-349_, lacking a trimerization domain, retains some processivity (133 ± 14), its *P*^*intr*^ is reduced compared to wild-type (2400 ± 260) indicating that oligomerization does appear to contribute to TarS processivity. It is important to note that the *k*_*off*_ measurements were nevertheless performed on a complicated system, where TarS itself is composed of multiple domains (catalytic domain, linker, C1, C2) that may take part in polyRboP acceptor binding, and that the acceptor itself is a heterogeneous polymer ranging in size from 9–11 kDa [31]. The additional C1 and C2 domains in the wild-type construct as such are likely to contribute to increased substrate avidity (accumulated strength of multiple affinities) compared to TarS_1-349_ that lacks these domains. The *k*_*off*_ values therefore would be expected to represent a myriad of interactions, all or some of which contribute to enzyme processivity. Furthermore, the method used here to analyze enzyme processivity is simplistic and only provides probabilistic estimates that are useful for comparative purposes only. More accurate measurements would require significantly more complicated studies that would address the genuine processivity of an enzyme acting on a polymeric substrate [60].

## Discussion

Gram-positive bacteria such as *S*. *aureus* invest an impressive amount of resources and energy in the synthesis of WTA, as these polymers play a pivotal role in bacterial biology and pathogenicity. In addition, the importance of accessory glycosyltransferases TarS and TarM in the decoration of *S*. *aureus* WTA polymers is becoming apparent and could provide valuable avenues for drug design. Here we have presented several structural snapshots of TarS that provide important insights into its catalytic mechanism.

The structure of TarS represents a unique topology, where an inverting GTA class GT2 family [32] catalytic domain is linked to a trimerization domain composed of tandem CBMs. The catalytic domain of TarS reveals interesting structural features, such as a CS loop that positions towards the catalytic center and a SA loop that becomes ordered upon donor substrate binding. We propose that these rearrangements act to limit the level of background hydrolysis while still allowing access of the incoming UDP-GlcNAc donor and release of the UDP leaving group. The above rearrangements may also guide the correct route of entry and assist in the binding of the polyRboP acceptor substrate. Interestingly, when superimposed, the structure of UDP bound SpsA (*Bacillus subtilis*) resembles the UDP (vs. UDP-GlcNAc) bound TarS catalytic GTA domain with regard to the position of the CS loop and the disorder of the SA loop (S6 Fig). As SpsA is an inverting GTA class GT2 family glycosyltransferase implicated in polysaccharide spore coat synthesis, and as most GT2 family members are also involved in polysaccharide synthesis or modification, the described rearrangements in the GTA domain may represent general mechanistic features of this family of enzymes acting on glycopolymer acceptor substrates. Similar loop rearrangements are observed in several other GTA families (reviewed in [61,62]). The structure of the trimerization domain of TarS is novel in the teichoic acid pathway, in that it includes two sequence-unique tandem CBMs, of which there are currently 71 classified families [32]. These tandem CBMs were identified by their high structural homology to the N1 (CBM68) and N2 (CBM48) domains of *Anoxybacillus sp*. LM18-11 pullulanase [35]. Pullulanase is a debranching enzyme that hydrolyzes α-1,6 glycosidic linkages of α-glucan polysaccharides, and its N1 domain exhibits sugar binding activity, such that the homologous C2 domain of TarS may have similarly been adapted for polyRboP binding. Although the N2 domain in the pullulanase structure is not observed as a complex, there are examples of CBM48 family structures in complex with oligosaccharides, including the rice branching enzyme 1 [63] and the starch excess4 protein [64], where the respective maltopentaose and maltoheptaose substrates interact with both the CBM48 and catalytic domains. Given the prominent electropositive charge distribution along the surface of the TarS C1 and C2 domains leading into the active site, it is likely that both CBM domains may be involved in polyRboP binding. Unlike pullulanase, the CBMs of TarS are involved in the formation of an extensive trimerization interface and interestingly, the truncation of both the TarS trimerization domain as well as the pullulanase N1 domain has only minimal effects on binding kinetics [35]. This suggests that these CBM domains are dispensable for catalytic activity and that they may assume other functions such as those involved in regulation and/or interaction. Indeed the TarS_1-349_ truncation mutant is capable of WTA GlcNAcylation albeit with a lower binding affinity for the polyRboP acceptor substrate (Fig 5D). Despite its structural divergence with TarS, a similar observation was made with TarM, whose GlcNAcylation activity appeared to be independent of the trimeric state of the enzyme as observed with a confirmed trimerization-disrupting mutant [31]. Furthermore, the TarS_1-349_ truncation mutant displayed a lower level of intrinsic processivity relative to wild-type, indicating that the trimerization domain does appear to play a role in enzyme processivity (Fig 5D). Another interesting observation is that although TarS and TarM are tasked with the similar undertaking of polyRboP GlcNAcylation, their trimerization domains differ dramatically in both structure as well as the extent of the trimerization interface, with TarM forming only sparse contacts. In fact, the trimerization domain of TarM belongs to pfam database [65] family domain of unidentified function (DUF) 1975, present in the N-termini of various prokaryotic α-glycosyltransferases. TarM in addition shows close structural resemblance to *Streptococcus pneumoniae* GtfA, a GTB O-GlcNAc transferase that is involved in the glycosylation of serine rich repeat adhesion proteins [66]. The DUF1975 domain of GtfA has been crystallographically observed to interact with a similar extended β-sheet domain of its GtfB co-activator, forming a hetero-tetramer [67]. As such, the vast difference in the derivation of the TarS and TarM trimerization domains supports the notion of separate evolutionary lineages and, along with other differences, elicits curiosity regarding the respective biological roles of these distant yet seemingly redundant enzymes.

An exciting study has recently reported the discovery of a novel antibody-antibiotic conjugate (AAC) for targeted killing of intracellular MRSA, which is strictly activated after the release of the antibiotic, rifalogue, in the proteolytic environment of the phagosome [68]. Interestingly, in designing the antibody-antibiotic conjugate, the authors found that an antibody that recognized the TarS-mediated β-O-GlcNAc WTA modification bound to all tested *S*. *aureus* strains, whereas the antibody recognizing the α-O-GlcNAc modification did not (presumably due to the absence of the α-O-linkage in specific *S*. *aureus* isolates) [68]. With regard to *in vivo* derived MRSA strains that co-produce the β- and α-O-linked GlcNAc WTA, antibodies specific for β-O-GlcNAc consistently yielded greater binding, suggesting that the TarS-mediated β-O-GlcNAc modification is either more immunogenic or abundant in MRSA pathogens. In support of this notion, it has also been found that human sera predominantly contain antibodies against β-O-GlcNAcylated WTA during infection, possibly highlighting the role of TarS in pathogenesis [69]. TarS rather than TarM has furthermore been shown to be key in MRSA β-lactam resistance [5], where β-O-GlcNAc is proposed to act as a possible scaffold for the recruitment of the β-lactam insensitive PBP2a, based on *in vitro* WTA binding of PBP2a [29]. Interestingly, in contrast to TarS, the TarM gene resides chromosomally outside of the WTA gene cluster, and its phylogenic distribution in Staphylococci suggests its acquisition by an ancient horizontal gene transfer (HGT) event [70]. In addition, whereas nearly all *S*. *aureus* strains contain the gene for TarS, several strains appear to have lost the TarM gene during evolution [70]. The retention of TarM in the genomes of a wide variety of *S*. *aureus* strains nevertheless suggests an advantage for its bacterial hosts. A recent article has shown that whereas TarS mediated β-O-GlcNAcylation of polymers facilitates susceptibility to infection by *Podoviridae* “lytic” phages, the TarM mediated α-O-GlcNAcylation prevents infection [70], thereby conferring a level of protection that likely evolved in *Podoviridae*-rich environments. The authors also report that *S*. *aureus* strains that express both TarS and TarM display preferential α-O-GlcNAcylation of polyRboP, leading them to suggest the possibility of TarM having a greater activity [70]. In accordance with this hypothesis, we found that TarM does appear to be a more catalytically efficient enzyme *in vitro*, possessing a ~10 fold greater second order rate constant *k*_*cat*_/K_m_ [31]. Redundancy in WTA glycosyltransferases TarS and TarM may also be beneficial for phage-mediated HGT events that drive *S*. *aureus* evolution, where a recent study has demonstrated the binding of “helper” phages to O-GlcNAc residues of *S*. *aureus* WTA, regardless of linkage conformation [71]. This redundancy in addition appears to be relevant in other processes such as colonization, where it has recently been found that WTA O-GlcNAcylation with either an α or β linkage is pivotal to the attachment of MRSA strains to human nasal epithelial cells [72], a process that contributes to the spread of nosocomial and community-acquired infections.

Although WTA is involved in a vast number of pathological processes in *S*. *aureus*, it has been shown that its absence nevertheless results in a viable phenotype. Bacteria lacking WTA are however greatly compromised in their ability to colonize and infect, and display dysregulated cell-division [5,10,73]. The deletion of TarS, TarM, or both, resulting in non-GlcNAcylated WTA, however leads to no detectable morphological abnormalities [5]. Therefore disruption of TarS in MRSA strains (especially those also lacking TarM) would interfere with host colonization, effectively disarming the pathogen without creating a strong selective pressure that often results in drug resistance. Furthermore, in MRSA strains where TarS is involved in methicillin resistance, inhibition of TarS in combination therapies would allow re-sensitization of bacteria to β-lactam antibiotics [5]. Indeed the success of β-lactam and β-lactamase inhibitor combinations in overcoming resistance attests to the effectiveness of such therapeutic strategies [74]. On another front, as TarS and TarM mediated WTA O-GlcNAcylation influences phage binding, the study of these enzymes will likely prove important in the re-emerging field of phage therapy [75]. The structure and mechanism of TarS therefore provide a valuable platform for rational therapeutic design in the treatment of MRSA, which remains a leading source of drug-resistant and life-threatening infections world-wide.

## Materials and Methods

### Cloning and protein synthesis

The full-length open reading frame (amino acids 1–573) encoding *S*. *aureus* TarS (SAV0258) was cloned into the expression vector pET41b without an affinity tag. Mutagenic TarS constructs were produced with the Quick Change mutagenesis kit (Qiagen). Constructs were transformed into Rosetta (DE3) *Escherichia coli*. The TarS truncation mutant (TarS_1-349_) was cloned into the expression vector pET41b with a C-terminal 6x His-tag. Protein expression was carried out overnight at 30°C. Cells were grown in Luria Bertani broth (supplemented with 35 μg/mL kanamycin) to an optical density (600 nm) of 0.6–0.8, at which point Isopropyl β-D-1-thiogalactopyranoside (IPTG) was added at a final concentration of 1 mM. Cells were pelleted and stored at -80°C until required.

### Protein purification

For purification of full-length TarS, cell pellets were resuspended in buffer A (20 mM Hepes pH 7.3, 300 mM NaCl, 5% glycerol). A complete protease inhibitor tablet at 1x final concentration (Roche) and DNAse 1 at 1 μg/mL final concentration were added and cells were lysed at 12,000 psi using a French press (Thermo Electron Corporation). Cell debris was pelleted by centrifugation at 20,000 x g for 30 min. The resulting supernatant was loaded onto a 5 mL Heparin HP cartridge (GE Lifesciences) and eluted over a 30 mL linear gradient to 100% buffer B (20 mM Hepes pH 7.3, 2 M NaCl, 5% glycerol). Fractions containing the purest protein were pooled, concentrated and loaded on a Superdex 200 column (GE Lifesciences) equilibrated in buffer C (20 mM Hepes pH 7.5, 500 mM NaCl, 5% glycerol) and the fractions collected and concentrated. For purification of the TarS truncation mutant (TarS_1-349_), the cell lysate was produced as above (but resuspended in buffer D: 20 mM NaPO_4_ pH 7.3, 5% glycerol), was loaded onto a 1 mL HisTrap HP cartridge (GE Lifesciences) and eluted over a 30 mL linear gradient to 100% buffer E (20 mM NaPO_4_ pH 7.3, 500 mM imidazole, 5% glycerol). Fractions containing the purest protein were pooled, concentrated and loaded on a Superdex 200 column (GE Lifesciences) equilibrated in buffer C and the fractions collected and concentrated. The protein was frozen in liquid N_2_ and stored at -80°C until required.

### Metal binding analysis

The metal content of TarS was measured using an inductively coupled plasma mass spectrometer (NexION 300D ICP-MS, PerkinElmer Life Sciences) and the data analyzed with NexION software. A calibration standard (CAT# IV-STOCK-4, Inorganic Ventures) containing metals of interest (Mg^2+^, Mn^2+^, Co^2+^, Ni^2+^, Cu^2+^, Zn^2+^) was diluted with an internal standard solution containing 10 μg/L Sc and 1% nitric acid (CAT# IV-ICPMS-71D, Inorganic Ventures), and this was used to generate standard curves spanning 1 to 100 μg/L for each metal. Protein samples were appropriately diluted with internal standard solution to adjust metal concentrations within the range of the standard curve. To confirm the absence of cation, the protein sample was spiked with metals of interest and measured as a positive control.

### WTA isolation and purification

*S*. *aureus* WTA was isolated and purified according to modifications of previously established protocols [76,77]. *S*. *aureus* RN4220 cells were grown in a culture of 20 mL TSB overnight at 37°C and the cells collected at 2,000 x g for 10 min. The cells were washed once in 30 mL of buffer 1 (50 mM MES, pH 6.5), resuspended in buffer 2 (4% SDS, 50 mM MES, pH 6.5), and boiled in a water bath for 1 hr. The cell debris was collected at 10,000 x g for 10 min, resuspended in 2 mL of buffer 2, and sedimented at 14,000 x g for 10 min. The pellet was washed in subsequent 1 mL volumes of buffer 2, buffer 3 (2% NaCl, 50 mM MES, pH 6.5), and buffer 1. The pellet was resuspended in 1 mL of buffer containing proteinase K (20 mM TrisHCl, pH 8.0, 0.5% SDS, 20 ug proteinase K) and digested at 50°C for 4 hr. The sample was pelleted at 14,000 x g for 10 min and washed once in buffer 3 and three times with distilled H_2_O. The sample was then resuspended in 1 mL of 0.1 M NaOH and shaken at room temperature for 16 hr. The remaining insoluble cell wall debris was removed by centrifugation at 14,000 x g for 10 min and the supernatant containing the hydrolyzed crude WTA was neutralized with addition of HCl to a final concentration of 0.1 M. The sample was dialyzed against distilled H_2_O using a 3 MWCO membrane. For digestion of attached GlcNAc, the sample was exchanged into buffer (100 mM Sodium Citrate pH 4.5, 250 mM NaCl) by dialysis using a 3 MWCO membrane and incubated with 0.5 mg/mL α-N-acetylglucosaminidase (R&D systems) and 0.2 mg/mL β-N-acetylglucosaminidase (New England Biolabs) overnight at 37°C. For purification, the sample was exchanged into buffer A (20 mM Tris HCl pH 7.2) by dialysis using a 3 MWCO membrane, applied on a 5 mL DEAE FF cartridge (GE Lifesciences) and eluted over a 30 mL linear gradient to 100% buffer B (20 mM Tris-HCl pH7.2, 1 M NaCl), with UV monitored at 205 nm. Peaks with the highest 205 nm readings were pooled and dialyzed against distilled H_2_O using a 3 MWCO membrane. The sample was frozen, lyophilized, and resuspended in distilled H_2_O. WTA concentration was measured according to the concentration of phosphorus detected by ICP-MS, whereby a phosphorus calibration curve spanning 1 to 100 μg/L was created using a phosphate standard solution (Sigma) diluted with internal standard solution (see metal binding analysis section).

### In vitro activity assay

TarS activity was studied using the ADP Quest Assay kit (DiscoverRx, USA) according to the manufacturer’s protocol and performed in 10 μL volume 384-well black assay plates. Various concentrations of wild-type and TarS_1-349_ were incubated with 1 mM UDP-GlcNAc and assay kit reagents to determine the optimal concentration of proteins for assay. Upon cleavage of UDP-GlcNAc by TarS, UDP is released resulting in a fluorescence signal monitored continuously at 530 nm excitation and 590 nm emission wavelengths using a Synergy H4 multi-mode plate reader (BioTek, USA). K_m_ and *k*_*cat*_ values were determined by using optimal protein concentrations and varying concentrations of UDP-GlcNAc and WTA, and concentration units were obtained using UDP for the standard curve. TarS activity was further verified directly by chromatography. Here, 10 μM TarS was incubated overnight with 1 mM UDP-GlcNAc, after which the reaction mixture was filtered through a 3 KDa MWCO filtration unit to remove protein. 10 ul fractions of the filtrate were injected onto a TSKgel DEAE-5PW weak anion exchange column (TOSOH Biosciences, USA) and the separated UDP-GlcNAc and UDP peak areas were monitored and quantified at a UV wavelength of 254 nm using an HPLC system.

### Thermostability analysis

TarS thermostability was measured as a function of temperature dependent aggregation by differential static light scattering (StarGazer-2; Harbinger Biotechnology and Engineering Corporation) according to the method of Vedadi et al. [78]. Briefly, 10 μl of 10 μM protein under selected conditions was heated from 25–85°C at a rate of 1°C/min in individual wells of a clear-bottom 384 well plate (Nunc, Rochester, NY). Protein aggregation, as a measure of the intensity of scattered light, was scanned every 30 s with a CCD camera. The integrated intensities were plotted against temperature using a Boltzman regression, where the inflection point of each fitted curve was defined as the aggregation temperature, T_agg_.

### Crystallization and structure solution

Crystals of TarS_217-573_ were obtained by proteolytic cleavage of ~10 mg/ml of the full-length protein with thermolysin (1 μg/ml final concentration) and immediate setup by vapor diffusion using a reservoir solution of 1 M imidazole (pH 7). Crystals belonged to space group P32 with unit cell dimensions a = b = 105.66 Å, c = 80.48 Å. For phasing, crystals were soaked in sodium iodide and a single wavelength SAD experiment was carried out. Data were processed with XDS [79] and Aimless [80]. Phasing was carried out with SHARP [81] using SHELX [82] to determine the heavy atom substructure. Model building was performed with Buccaneer [83] and refined using Refmac [84], Phenix [85] and Coot [86] using TLS parameters in the later stages. Refinement was finished using native data to higher resolution (see Table 1). The final model with one molecule in the asymmetric unit has good stereochemistry with 97% of residues in the favoured region of the Ramachandran plot and 0% outliers.

Crystals of TarS_1-349_ (~20 mg/mL) were obtained by sitting-drop vapor diffusion in the presence (or absence) of 15 mM UDP-GlcNAc and 2 mM MnCl_2_ using a reservoir solution of 0.2 mM lithium sulfate, 27% w/v PEG 3350, and 0.1 M Bis-Tris pH 5.5. Crystals were cryo-protected with a reservoir solution containing 30% glycerol and 50 mM UDP-GlcNAc when required and were subsequently flashed cooled. Crystals belonged to space groups C2 (native), P1 (+UDP-GlcNAc:Mn, +UDP-GlcNAc) or P21 (+UDP) with the same general molecular packing (see Table 1). The structures were solved with molecular replacement using Phaser [87], initially using the overlapping region of the TarS_217-573_ structure as a search model and refined as above. The final models have good stereochemistry: the native structure has one monomer in the asymmetric unit with 97% of residues in the favoured region of the Ramachandran plot and 0.6% outliers; the UDP-GlcNAc:Mn structure has two monomers in the asymmetric unit with 97% of residues in the favoured region of the Ramachandran plot and 1.4% outliers; the UDP structure has one monomer in the asymmetric unit with 97% of residues in the favoured region of the Ramachandran plot and 0.3% outliers; and the UDP-GlcNAc structure has two monomers in the asymmetric unit with 98% of residues in the favoured region of the Ramachandran plot and 0.29% outliers.

Full-length TarS (~20 mg/mL) was crystallized by microbatch in the presence of 15 mM UDP-GlcNAc and 2 mM MnCl_2_ using a reservoir solution of 0.2 M ammonium chloride, 15% w/v PEG 6000, 0.15 M Tricine (pH 8.0), and 0.4 M NDSB 195. Crystals were cryo-protected with a reservoir solution containing 30% glycerol and 15 mM UDP-GlcNAc and were subsequently flashed cooled. Crystals belonged to space group P21 with unit cell dimensions a = 71.85 Å, b = 191.47 Å, c = 97.03 Å, β = 109.5°. Crystals diffracted with strong anisotropy (accounting for poor dataset statistics) and the data were cut to 4.0 Å. The structure was solved with molecular replacement using Phaser [87] with TarS_1-349_ and TarS_217-573_ structures as search models. The structure was refined with Phenix [85] using the high-resolution structures as restraints. The final model with three molecules in the asymmetric unit has good stereochemistry with 96% of residues in the favoured region of the Ramachandran plot and 0.24% outliers.

Data and coordinates have been deposited to the RCSB Protein Data Bank with accession codes 5TZ8, 5U02, 5TZI, 5TZK, 5TZE and 5TZJ.

### Analysis of quaternary structure

Purified protein was applied to a Superdex 200 HR 10/30 column (GE Healthcare) equilibrated in 30 mM Hepes pH 7.3, 500 mM NaCl, 5% glycerol buffer, using an Agilent 1100 series HPLC (*Agilent* Technologies), coupled in-line to a Dawn Heleos II 18-angle MALS light scattering detector, and Optilab T-rEX differential refractometer monitor (Wyatt Technology). Monomeric bovine serum albumin (Sigma-Aldrich) was used to normalize the light scattering detectors. Data were collected and analyzed with the Astra 6 software package provided by the manufacturer, Wyatt Technology. The protein molar mass was calculated, assuming a refractive index increment (dn/dc) value of 0.186 ml g^−1^.

### LC/MS analysis of TarS RboP-GlcNAc product

Reactions in the presence of 50 μM TarS, 8 mM UDP-GlcNAc and ± 8 mM RboP proceeded overnight at room temperature in buffer consisting of 20 mM Tris pH 8, 500 mM NaCl, and 2 mM MnCl_2_. Reactions were subsequently filtered through a 3 KDa MWCO filtration unit to remove protein. In order to remove buffer and salts for LC/MS analysis, samples were diluted 50 fold in distilled H_2_O, applied to a HiTrap Q XL 1mL cartridge and eluted with 200 mM ammonium formate pH 8. The eluted fraction was extensively diluted with water and repeatedly lyophilized to reduce salt concentration. The lyophilized sample was dissolved in water or water/MeOH (1:1) and dilution series were subjected to LC/MS. Spectra were recorded on a Waters ZQ2000 LC/MS attached to a Waters 2695 separation module with flow injection analysis in negative mode using electrospray ionization. Spectra were analyzed with Masslynx 4.0 software.

### Determination of association/dissociation rate constants

Biolayer interferometry was performed using an Octet Red instrument (FortéBio Inc.) with streptavidin sensors (FortéBio Inc.). TarS was biotinylated using EZ-Link NHS-PEG4-Biotin (Thermo Scientific, USA). Biolayer interferometry was performed at 25°C in a 96-well plate (Greiner Bio-One) and a 200 μL well volume. After a brief equilibration of the sensors in assay buffer (20 mM Hepes pH 7, 500 mM NaCl), full-length or TarS_1-349_ was loaded onto sensors for 5 minutes at 300 nM followed by the blocking of unbound streptavidin with 15 μg/mL EZ-Link Biocytin (Thermo Scientific) in Superblock Blocking Buffer (Thermo Scientific). Next, a baseline was acquired for 3 minutes followed by the association of TarS for 5 minutes (*k*_*on*_) and dissociation for 15 minutes (*k*_*off*_) in assay buffer (20 mM Hepes pH 7, 500 mM NaCl). Various optimal concentrations of polyRboP (0.31 mM, 0.62 mM, 1.25 mM, 2.5 mM and 5 mM) were titrated with double referencing to rule out non-specific binding to sensors, and the K_D_ was calculated based on *k*_*on*_ and *k*_*off*_ rates fitted to a heterogeneous ligand model using the FortéBio data analysis software.

## Supporting Information

S1 Fig Topology diagram. Secondary structural elements are presented, where α-helices are indicated by pink arrows and β-strands by red cylinders. The respective domains (with encompassing amino acids) are demarcated by background color. Ribbon representation of a full-length TarS monomer is also provided below and colored according to the topology diagram. The regions of TarS encompassed by the TarS_1-349_ and TarS_217-573_ structures are also indicated, with missing regions displayed in grey. The topology diagram was generated with PDBSum.
(TIF)

S2 Fig Comparison of TarS_1-349_ structures. (A) Overlay of the ribbon representation of various TarS_1-349_ structures in complex with Mn^2+^ (green), Mn^2+^/UDP (beige), UDP-GlcNAc (pink), and Mn^2+^/UDP-GlcNAc (blue). (B) Close up of ligands as described in (A). Ligands are displayed in stick form, colored according to heteroatom type, and correspond in color to respective structures. Mn^2+^ are represented as purple spheres and the locations of the SA and CS loops indicated. (C) mFo-dFc simulated annealing omit maps for UDP bound in full-length and TarS_1-349_ structures and UDP-GlcNAc bound in the TarS_1-349_ structure as indicated, generated with pymol and contoured at 2.5 sigma.
(TIF)

S3 Fig Validation of the full-length TarS structural data. (A) Composite OMIT map calculated with Phenix for the TarS full-length structure. The catalytic domain of chain A (gold), with few crystal contacts, has weaker electron density compared to chains B (cyan) and C (magenta). (B) 2mFo-dFc (blue) and mFo-dFc (green) density calculated after refinement of full-length TarS with residues 412–415 deleted in each chain (chains A,B,C = gold, cyan, magenta respectively). To minimize bias prior to refinement, model perturbation was carried out with phenix.dynamics and B-factors were reset to 10.
(TIF)

S4 Fig Comparison of Pullulanase and TarS structures. (left) Overlay of the N1 (yellow) and N2 (orange) domains of the *anoxybacillus* pullulanase onto the C2 and C1 domains of a TarS_217-573_ monomer. (right) The full structure of pullulanase is displayed alongside for comparison (PDB 3WDH), where N1 is in the same orientation as in the TarS (C2) superimposed domain.
(TIF)

S5 Fig SEC-MALS elution profile of full-length TarS. The protein was run at a concentration of 25 μM and a horizontal line corresponds to the molecular weight as listed.
(TIF)

S6 Fig Comparison of SpsA and TarS_1-349_ structures. (A) Overlay of the ribbon representation of SpsA (PDB:1qgq) in complex with UDP/glycerol (peach) and TarS_1-349_ in complex with UDP-GlcNAc (green) and UDP (blue) (only UDP-GlcNAc is displayed for simplicity). (B) Close up of ligands for UDP complexed SpsA and UDP-GlcNAc complexed TarS_1-349_ structures, as described in (A). Ligands are displayed in stick form and colored according to heteroatom type, and Interactions between atoms are displayed by dotted lines.
(TIF)

S7 Fig LC/MS of TarS reaction products. Representative LC/MS spectra of anion exchange resin enriched TarS reaction mixtures containing UDP-GlcNAc (m/z 605.9 and 628) in the absence (A) and presence (B) of ribitol-1-phosphate (RboP; m/z 231.1). The target compound, RboP-GlcNAc (m/z 434.1) was found only in the sample containing ribitol-1-phosphate.
(TIF)

S8 Fig Biolayer interferometry measurement of TarS-polyRboP. Association/dissociation curves were obtained by loading streptavidin sensors with biotinylated full-length or TarS_1-349_ constructs followed by titration with various concentrations of polyRboP (0.31 mM, 0.62 mM, 1.25 mM, 2.5 mM, and 5 mM).
(TIF)

S1 Movie Structural morph among full-length TarS trimer monomers. TarS trimer monomers were overlayed along the trimerization domain, illustrating possible motion of relative catalytic domains. UDP-GlcNAc (blue) is displayed in stick form and colored according to heteroatom type.
(MP4)
