# Supplementary figures and images for: Structure and Mechanism of Staphylococcus aureus TarS, the Wall Teichoic Acid β-glycosyltransferase Involved in Methicillin Resistance
Source: PLoS Pathog. 2016 Dec 14;12(12):e1006067. doi: 10.1371/journal.ppat.1006067 (PMC5156392; doi:10.1371/journal.ppat.1006067)

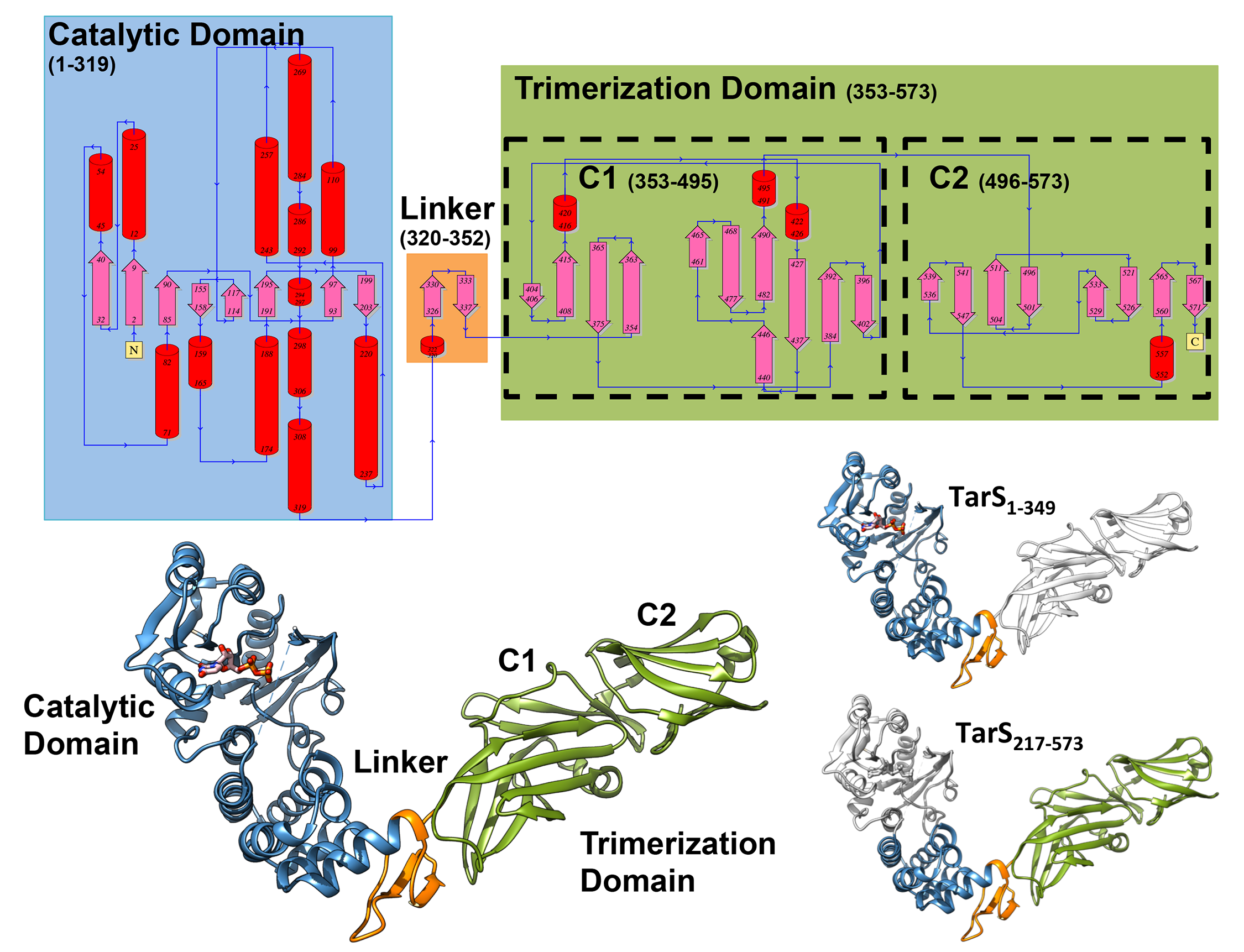

Supplement: S1 Fig — Secondary structural elements are presented, where α-helices are indicated by pink arrows and β-strands by red cylinders. The respective domains (with encompassing amino acids) are demarcated by background color. Ribbon representation of a full-length TarS monomer is also provided below and colored according to the topology diagram. The regions of TarS encompassed by the TarS1-349 and TarS217-573 structures are also indicated, with missing regions displayed in grey. The topology diagram was generated with PDBSum. (TIF) [file ppat.1006067.s001.tif]

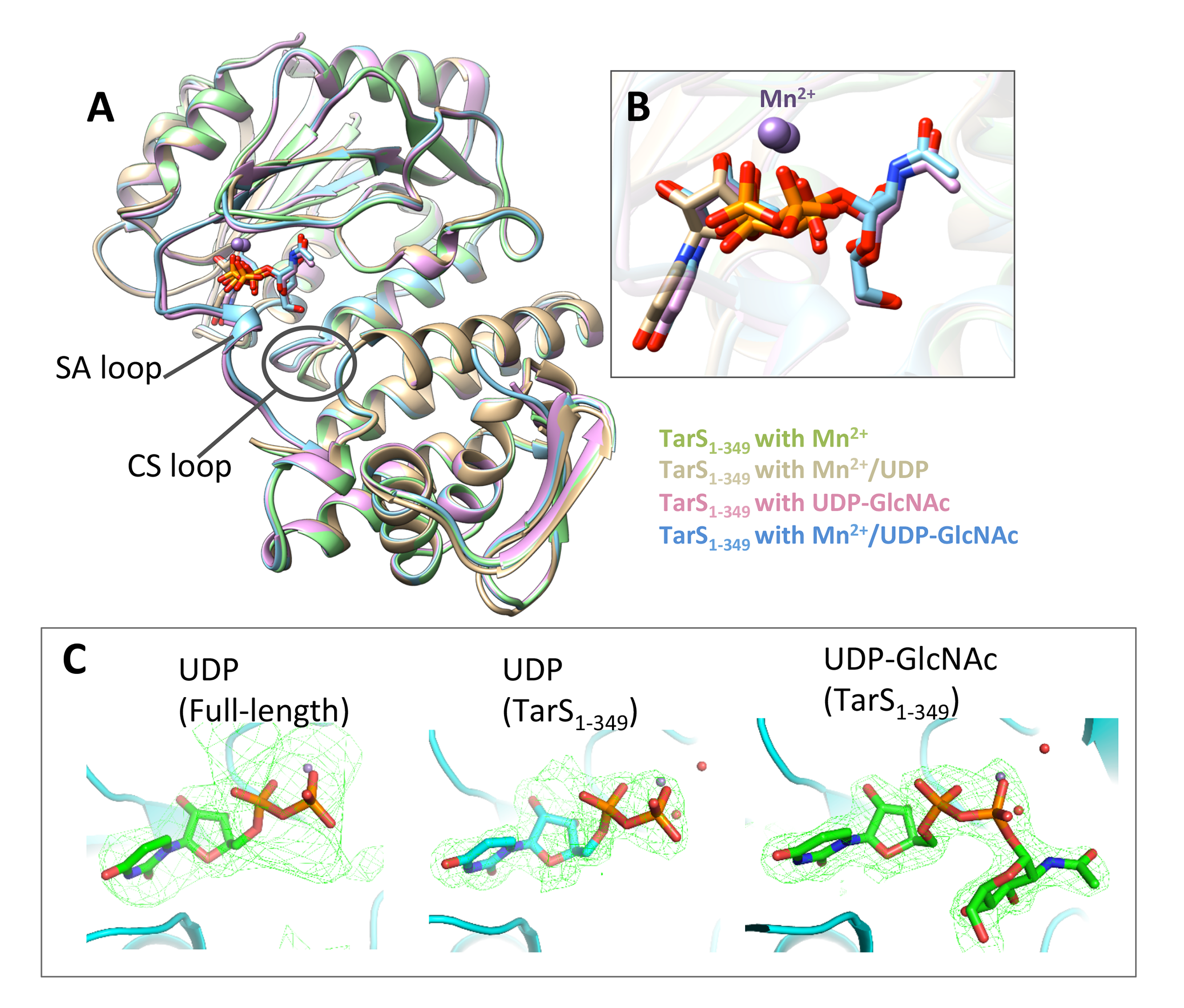

Supplement: S2 Fig — (A) Overlay of the ribbon representation of various TarS1-349 structures in complex with Mn2+ (green), Mn2+/UDP (beige), UDP-GlcNAc (pink), and Mn2+/UDP-GlcNAc (blue). (B) Close up of ligands as described in (A). Ligands are displayed in stick form, colored according to heteroatom type, and correspond in color to respective structures. Mn2+ are represented as purple spheres and the locations of the SA and CS loops indicated. (C) mFo-dFc simulated annealing omit maps for UDP bound in full-length and TarS1-349 structures and UDP-GlcNAc bound in the TarS1-349 structure as indicated, generated with pymol and contoured at 2.5 sigma. (TIF) [file ppat.1006067.s002.tif]

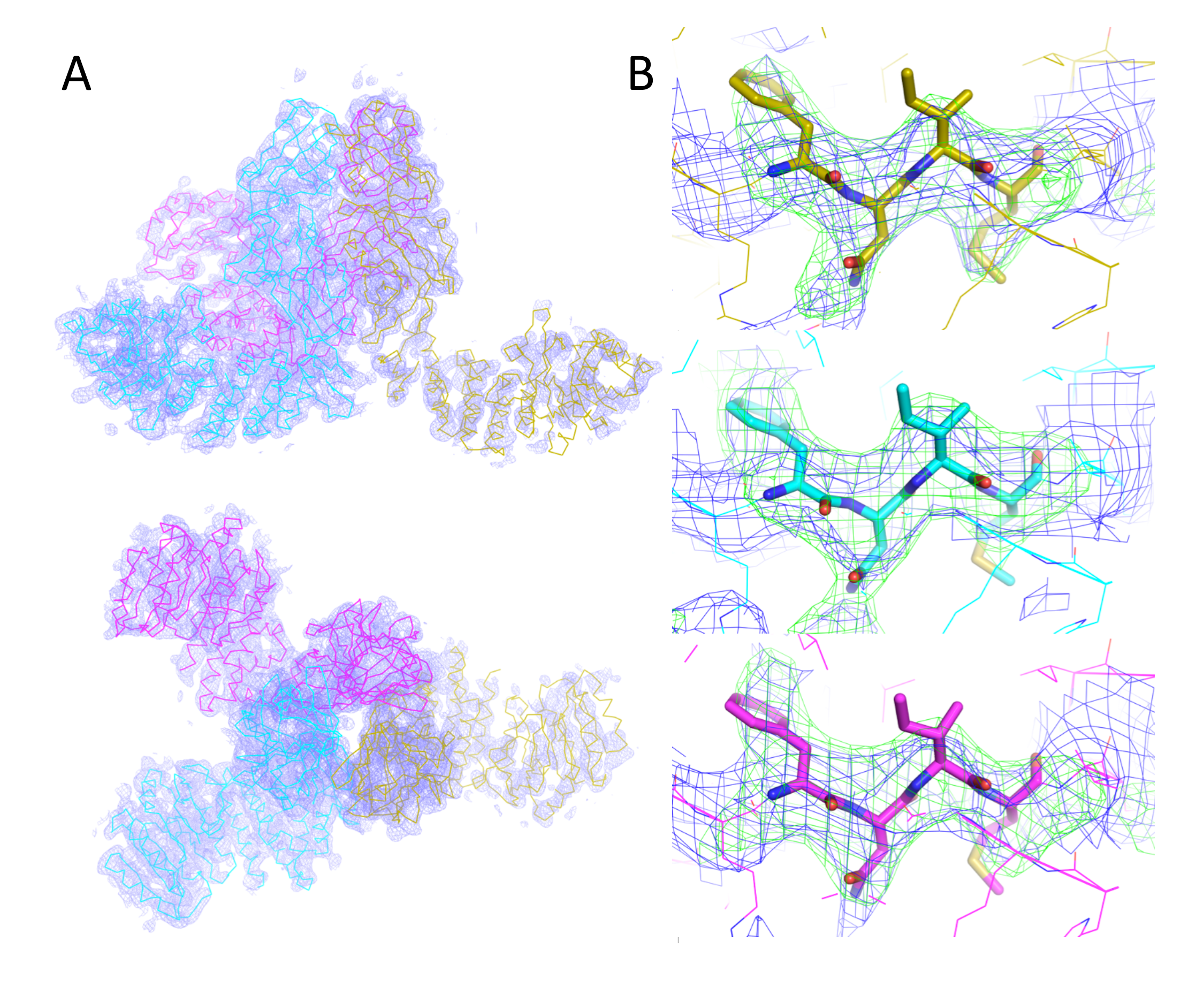

Supplement: S3 Fig — (A) Composite OMIT map calculated with Phenix for the TarS full-length structure. The catalytic domain of chain A (gold), with few crystal contacts, has weaker electron density compared to chains B (cyan) and C (magenta). (B) 2mFo-dFc (blue) and mFo-dFc (green) density calculated after refinement of full-length TarS with residues 412–415 deleted in each chain (chains A,B,C = gold, cyan, magenta respectively). To minimize bias prior to refinement, model perturbation was carried out with phenix.dynamics and B-factors were reset to 10. (TIF) [file ppat.1006067.s003.tif]

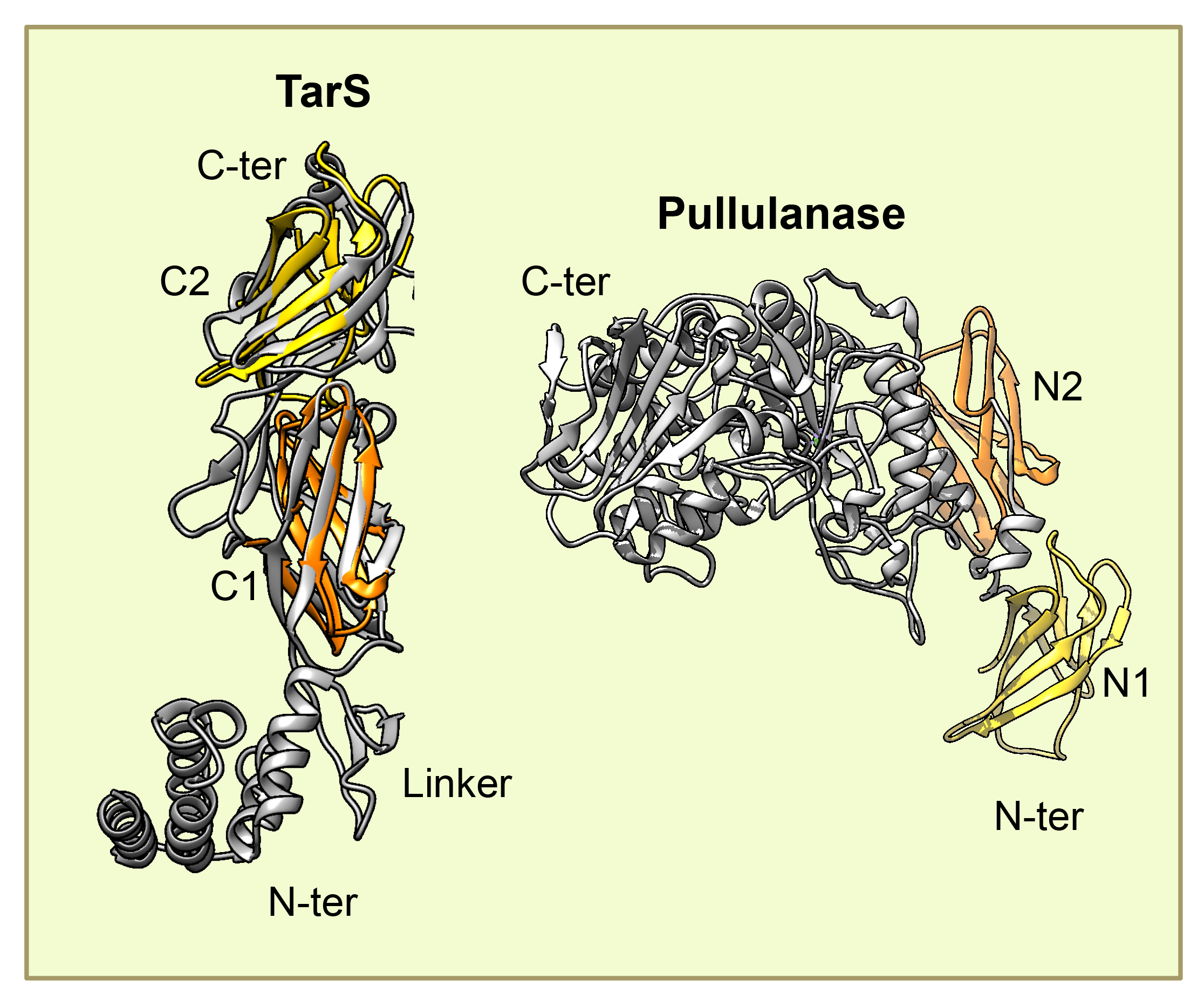

Supplement: S4 Fig — (left) Overlay of the N1 (yellow) and N2 (orange) domains of the anoxybacillus pullulanase onto the C2 and C1 domains of a TarS217-573 monomer. (right) The full structure of pullulanase is displayed alongside for comparison (PDB 3WDH), where N1 is in the same orientation as in the TarS (C2) superimposed domain. (TIF) [file ppat.1006067.s004.tif]

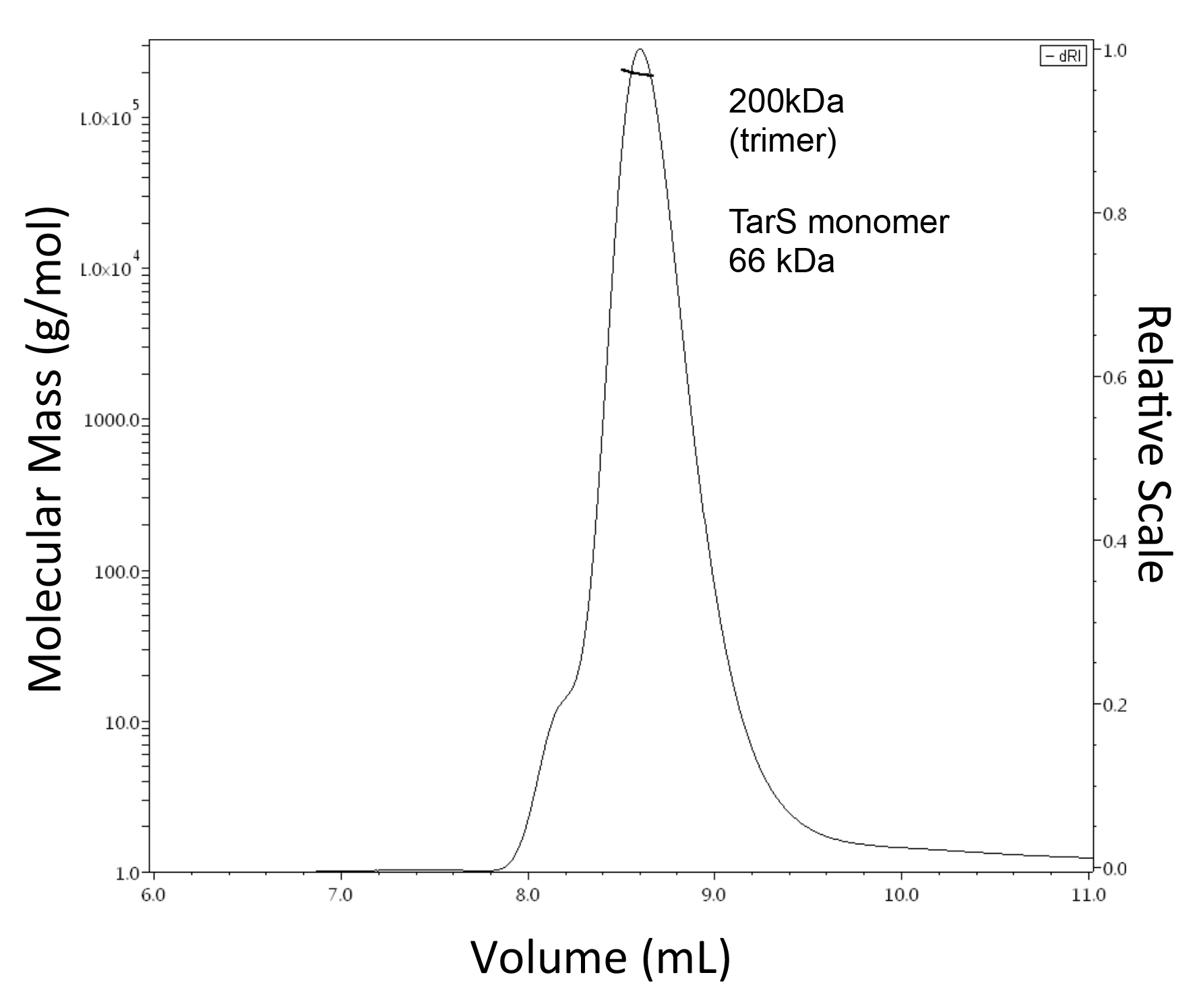

Supplement: S5 Fig — The protein was run at a concentration of 25 μM and a horizontal line corresponds to the molecular weight as listed. (TIF) [file ppat.1006067.s005.tif]

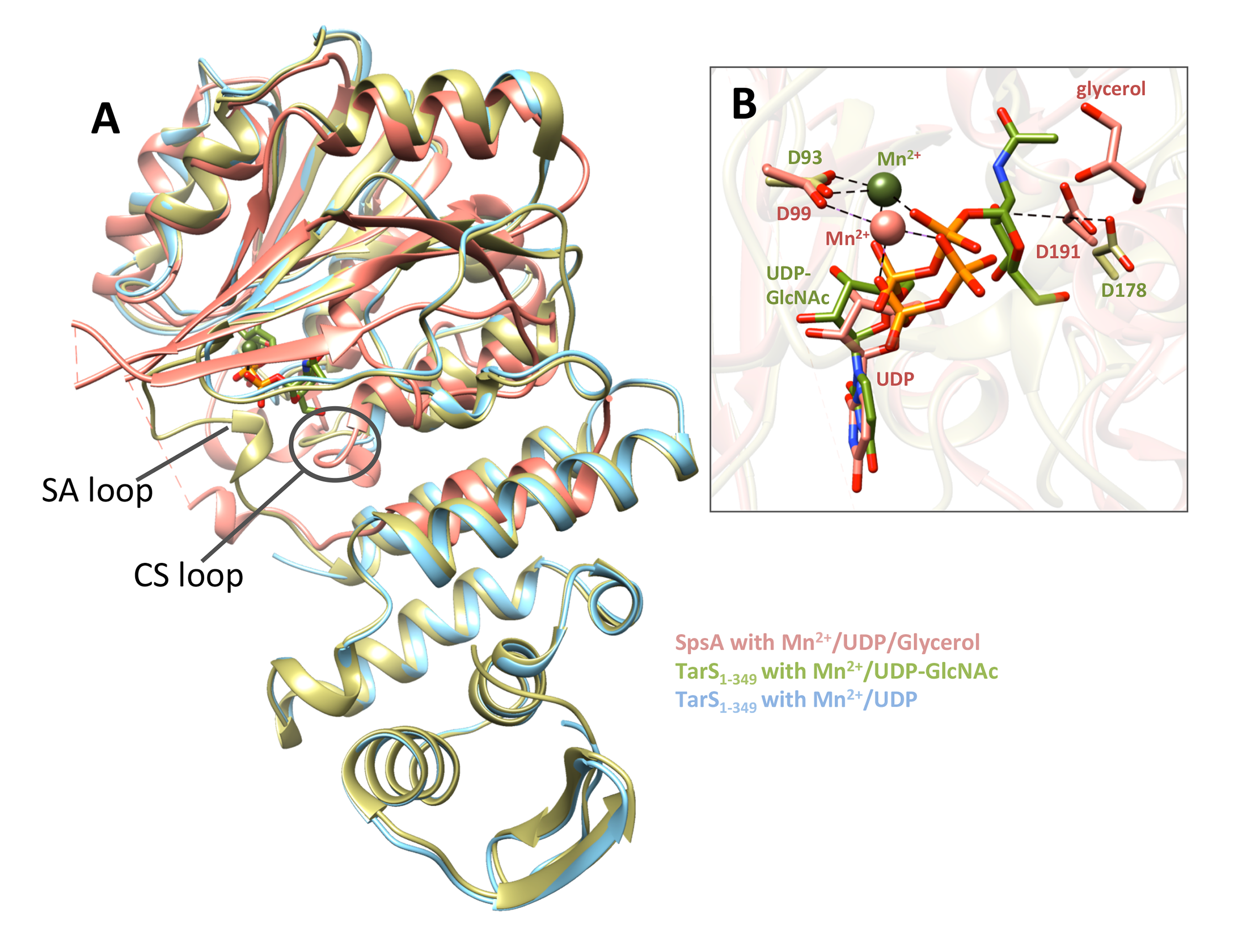

Supplement: S6 Fig — (A) Overlay of the ribbon representation of SpsA (PDB:1qgq) in complex with UDP/glycerol (peach) and TarS1-349 in complex with UDP-GlcNAc (green) and UDP (blue) (only UDP-GlcNAc is displayed for simplicity). (B) Close up of ligands for UDP complexed SpsA and UDP-GlcNAc complexed TarS1-349 structures, as described in (A). Ligands are displayed in stick form and colored according to heteroatom type, and Interactions between atoms are displayed by dotted lines. (TIF) [file ppat.1006067.s006.tif]

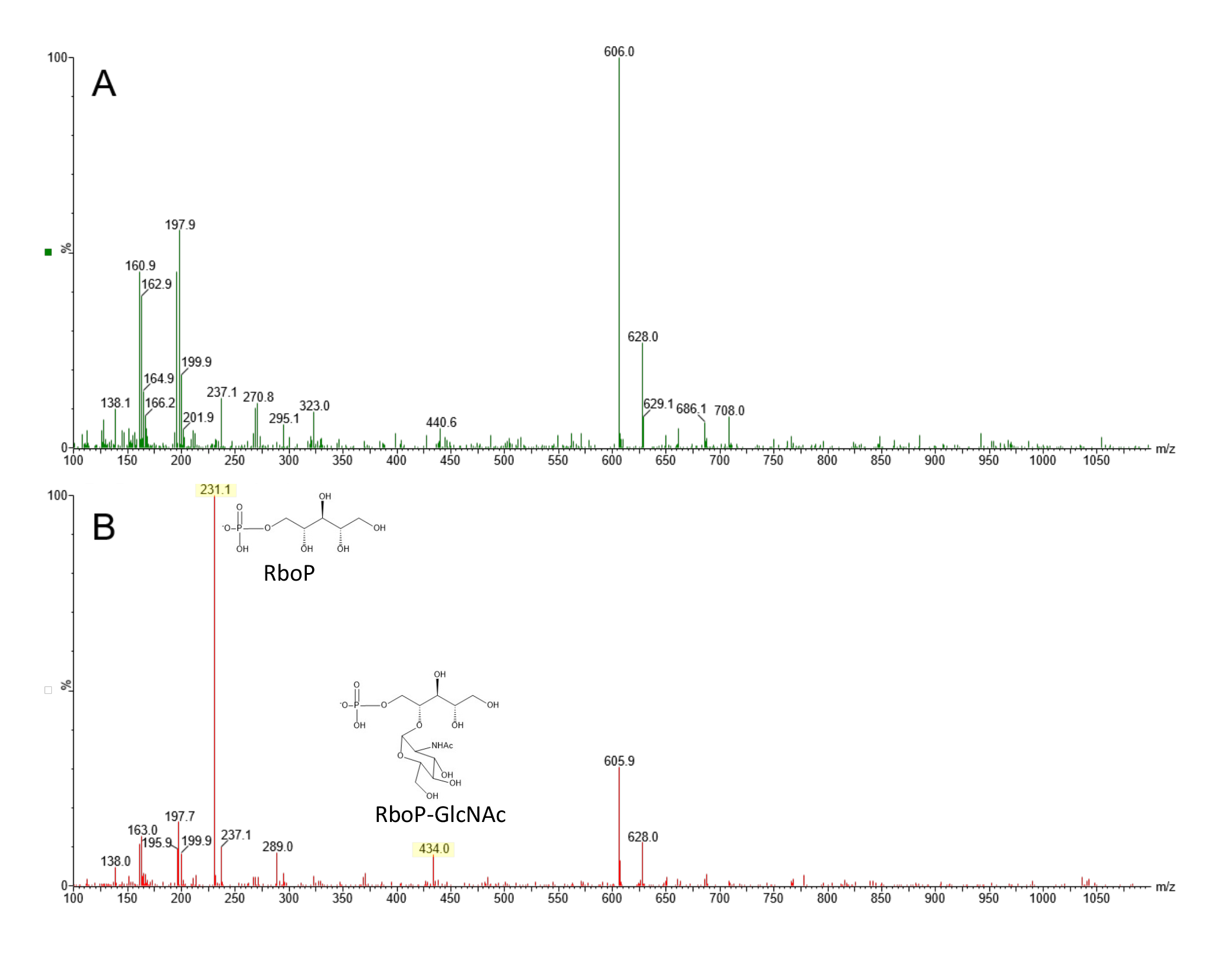

Supplement: S7 Fig — Representative LC/MS spectra of anion exchange resin enriched TarS reaction mixtures containing UDP-GlcNAc (m/z 605.9 and 628) in the absence (A) and presence (B) of ribitol-1-phosphate (RboP; m/z 231.1). The target compound, RboP-GlcNAc (m/z 434.1) was found only in the sample containing ribitol-1-phosphate. (TIF) [file ppat.1006067.s007.tif]

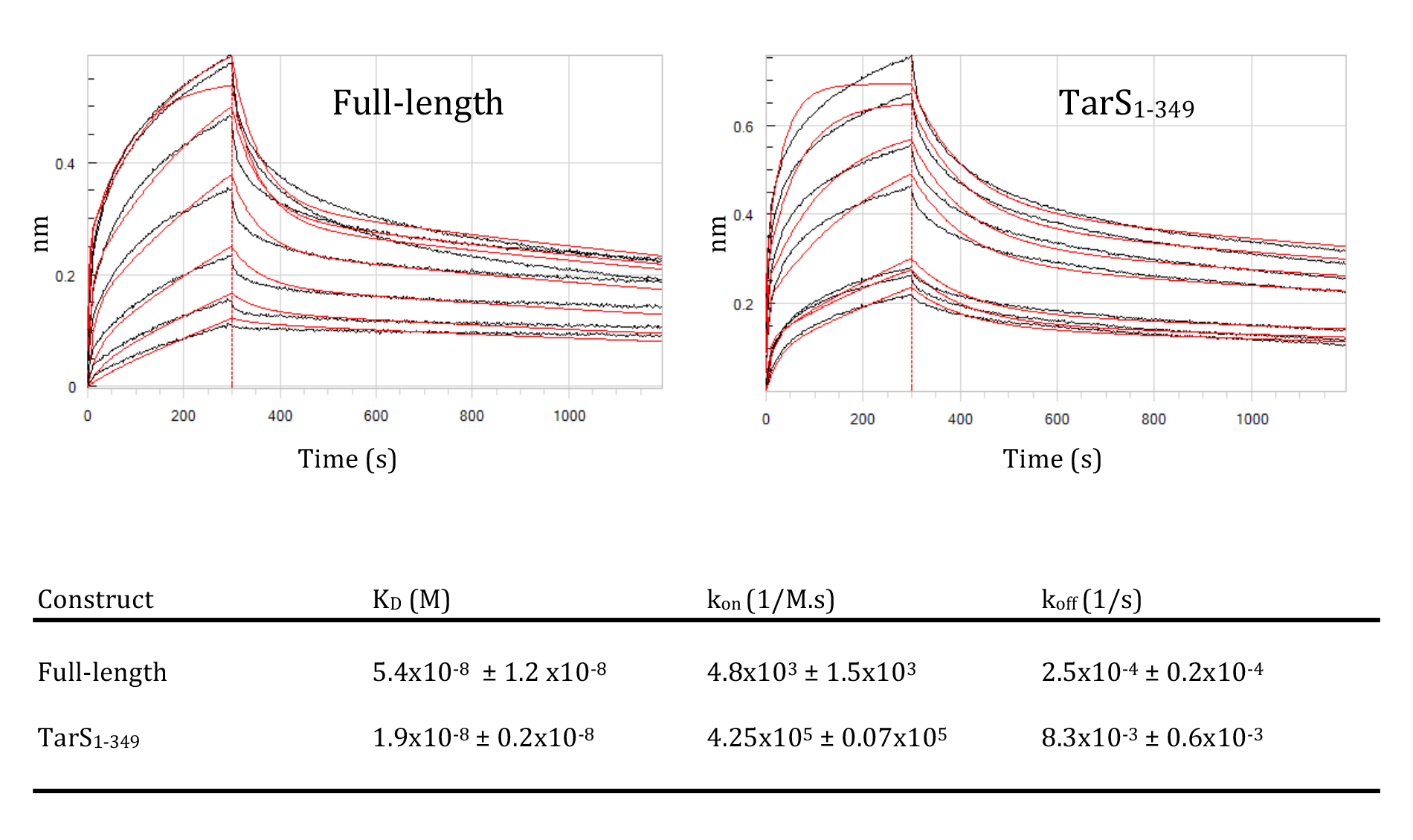

Supplement: S8 Fig — Association/dissociation curves were obtained by loading streptavidin sensors with biotinylated full-length or TarS1-349 constructs followed by titration with various concentrations of polyRboP (0.31 mM, 0.62 mM, 1.25 mM, 2.5 mM, and 5 mM). (TIF) [file ppat.1006067.s008.tif]
